# Exploring the link between dietary inflammatory index, inflammatory biomarkers, and sleep quality in adults with obesity: a pilot investigation

**DOI:** 10.1038/s41366-025-01728-2

**Published:** 2025-01-30

**Authors:** Hakan Toğuç, Hande Öngün Yılmaz, Bülent Yaprak

**Affiliations:** 1 Department of Nutrition and Dietetics, Faculty of Health Sciences, Inonu university, Malatya, Turkey; 2 Department of Nutrition and Dietetics, Faculty of Health Sciences, Bandırma Onyedi Eylül University, Balıkesir, Turkey; 3 Department of Internal Medicine, Malatya Training and Research Hospital, Malatya, Turkey

**Keywords:** Obesity, Risk factors, Inflammatory diseases

## Abstract

**Objective:**

Obesity is known to be associated with inflammation and impaired sleep quality. In addition, the anti-inflammatory properties of the daily diet provide positive effects on health. The aim of this study was to investigate the relationship between the inflammatory index of the diet consumed by people with obesity and inflammatory biomarkers and sleep quality.

**Method:**

This study included 124 people with obesity (F: 75; M: 49) with a mean age of 42.20 ± 11.00 years, who presented to a dietary outpatient clinic in Malatya between November 2021 and May 2022. Three-day dietary intake records were collected to calculate Dietary Inflammatory Index (DII) scores, which were then compared with inflammatory biomarkers, anthropometric measurements, and sleep quality measures.

**Results:**

Among the biochemical parameters, C-reactive protein (CRP) was found to be significantly higher in the groups with higher DII score (*p* = 0.006), and CRP (*r* = 0.258; *p* = 0.004) and total cholesterol (r = −0.243; *p* = 0.007) increased significantly with increasing inflammatory score of the diet. As the inflammatory burden of the diet consumed by the participants increased, their Body Mass Index (BMI) also increased (*p* = 0.009). No significant correlation was found between DII and sleep quality (*p* = 0.348).

**Conclusion:**

These findings suggest that an anti-inflammatory diet can effectively reduce inflammation and BMI in people with obesity, but has a limited effect on sleep quality. Therefore, it is recommended that dietitians integrate anti-inflammatory dietary strategies for people with obesity into their clinical practice.

## Introduction

Obesity, a multifaceted non-communicable condition, is characterized by an excessive energy balance, increased adipose tissue, and chronic low-grade inflammation [1]. The prevalence of obesity has alarmingly doubled globally over the last four decades, cutting across gender, age, socioeconomic status, and ethnicity [2]. This rising trend highlights the urgent need for novel strategies in obesity research, evaluation, management, and treatment [3].

Inflammation, a response to cellular injury, facilitates tissue repair and neutralizes harmful agents. It involves the release of various chemical mediators [4]. Persistent, unaddressed inflammation can lead to chronic conditions, contributing to diseases like type 2 diabetes, dyslipidemia, metabolic syndrome, cardiovascular diseases, and depression [5].

Recent studies have also linked inflammation to sleep quality. Inflammatory mediators, particularly cytokines, are crucial in the relationship between sleep and the immune system [6]. Research has shown a correlation between sleep disturbances and increased inflammatory markers [7]. Poor sleep quality can disrupt immune function, elevating markers like C-reactive protein (CRP) [8, 9].

The anti-inflammatory properties of various nutrients have been documented [10–12]. The Dietary Inflammatory Index (DII), developed by Shivappa et al. (2014), assesses the inflammatory potential of diets, based on an analysis of 1943 studies across 11 countries. This index, comprising 45 dietary components, categorizes diets as pro-inflammatory or anti-inflammatory [10].

Despite the expanding research on obesity treatment, there is a lack of studies on the impact of dietary inflammation on inflammatory markers and sleep quality in people with obesity. The aim of our pilot investigation was to explore the relationship between the inflammatory index of the diet consumed by adults with obesity and, inflammatory biomarkers, and sleep quality.

## Materials and Methods

This pilot investigation was conducted at the diet outpatient clinic of a state hospital in Malatya/Turkey from November 2021 to May 2022.

### Sample of the study

This study was conducted on 176 individuals (103 women, 73 men) who applied to the diet outpatient clinic of a public hospital in Turkey between November 2021 and May 2022. Based on the number of applicants, a programme called ‘Sample Size Calculator’ was used and the sample size was determined as 121 with a 95% confidence interval and a 5% margin of error. During the research process, 52 of the participants refused to participate in the study, resulting in 124 individuals successfully completing the study.

Eligibility for the study was confined to individuals aged 19–65 years with a Body Mass Index (BMI) of 30–40 kg/m^2^, free from chronic conditions such as diabetes, hypertension, dyslipidemia, cancer, or chronic renal failure. Exclusion criteria encompassed recent dietary program adherence, nutritional supplement intake, inflammatory disease diagnosis, or severe psychiatric disorders. Pregnancy and breastfeeding were also exclusion criteria. Essential for inclusion was voluntary participation without recent engagement in dietary interventions or supplement consumption.

### Data collection instruments

Data Collection Form: In this form consisting of four sections, socio-demographic information, sleep quality through Pittsburgh Sleep Quality Index and three-day dietary intake record were collected. It was administered through direct interviews. Dietary intake record and anthropometric measurements were collected by the researcher, while routine biochemical measurements were obtained from existing participant records.

#### Dietary intake records

To evaluate dietary patterns and calculate DII scores, participants recorded their food intake over three days, including one weekend day. Detailed instructions and examples were provided for accurate record-keeping.

#### Pittsburgh Sleep Quality Index (PSQI)

The PSQI, adapted for Turkish populations by Ağargün et al. (1996), assessed sleep quality over the past month. It includes 24 questions, with 19 self-reported and 5 completed by a spouse or roommate. Each item is scored from 0 to 3, with a maximum total score of 21. A score above five indicates poor sleep quality [13].

#### Biochemical measurements

Specific biochemical tests were routinely ordered for participants by an internist. These included fasting blood glucose, triglycerides, total cholesterol, LDL-cholesterol, and HDL-cholesterol. Additionally, inflammatory markers such as CRP, leukocyte, lymphocyte, monocyte, neutrophil, and eosinophil levels were measured. These parameters were analyzed using spectrophotometry on the Beckman Coulter AU640, with LDL cholesterol calculated using the Friedewald formula [14].

#### Computation of the Dietary Inflammatory Index (DII)

The DII, developed by Shivappa et al. (2014), evaluates the inflammatory potential of diets. It involves calculating z-scores for consumed nutrients, based on mean daily intakes and standard deviations established from national nutritional research datasets across various populations (Shivappa et al., 2014). The process entailed transforming z-scores to percentile scores, which were then adjusted (doubled and decremented by one) for symmetric distribution. These adjusted values were multiplied by customised effect scores for each nutrient. The DII is obtained by summing these values, representing the inflammatory potential of the diet [10]. In this study, data for 32 nutrient components was available for DII calculation. The customised effect scores, global average daily intakes, and standard deviation (SD) values pertinent to the DII computation for these nutrients are delineated in Table 1.

**Table 1:** Nutrient Parameters for Dietary Inflammatory Index Calculation: Customised Effect Scores, Global Average Daily Intakes and Standard Deviations (Shivappa et al. 2014).

Nutrients	Customised Full Impact Score	Mean Daily Global Intake	Standard Deviation
Energy (kcal)*	0.180	2056	338
Carbohydrate (g)	0.097	272.2	40
Protein (g)	0.021	79.4	13.9
Total fat (g)	0.298	71.4	19.4
Saturated fat (g)	0.373	28.6	8
Polyunsaturated fatty acids (g)	−0.337	13.88	3.76
Omega-3 fatty acids (g)	−0.436	1.06	1.06
Omega-6 fatty acids (g)	−0.159	10.8	7.5
Monounsaturated fatty acids (g)	−0.009	27	6.1
Cholesterol (mg)	0.110	279.4	51.2
Trans fat (g)	0.229	3.15	3.75
Vitamin B12 (μg)	0.106	5.15	2.7
Vitamin B6 (mg)	−0.365	1.47	0.74
β-carotene (μg)	−0.584	3718	1720
Caffeine (g)	−0.110	8.05	6.67
Dietary fiber (g)	−0.663	18.8	4.9
Folic acid (μg)	−0.190	273	70.7
Iron (mg)	0.032	13.35	3.71
Magnesium (mg)	−0.484	310.1	139.4
Niacin (mg)	−0.246	25.9	11.77
Riboflavin (mg)	−0.068	1.7	0.79
Selenium (μg)	−0.191	67	25.1
Zinc (mg)	−0.313	9.84	2.19
Thiamine (mg)	−0.098	1.7	0.66
Vitamin A (RE)	−0.401	983.9	518.6
Vitamin C (mg)	−0.424	118.2	43.46
Vitamin D (μg)	−0.446	6.26	2.21
Vitamin E (mg)	−0.419	8.73	1.49
Chilli (g)	−0.131	10	7.07
Garlic (g)	−0.412	4.35	2.9
Onion (g)	−0.301	35.9	18.4
Green/black tea (g)	−0.536	1.69	1.53

^*^(1 kcal = 4.186 kJ).

### Statistical analysis

Data analysis was conducted using IBM SPSS software (Version 22.0), with dietary consumption records assessed via the BeBIS 8.2 program. Descriptive statistics, including arithmetic mean, standard deviation, range, frequency, and percentage, were computed. DII scores were categorised into quartiles, with Q1 being the most anti-inflammatory and Q4 being the most pro-inflammatory.

The normality of data distribution was evaluated using histograms, Q-Q plots, and the Kolmogorov-Smirnov test. Categorical variables were analyzed with the Pearson chi-square test. The t-test and Analysis of Variance (ANOVA) were applied for independent group differences when parametric test assumptions were met. For non-parametric conditions, the Mann-Whitney U and Kruskal Wallis tests were employed. Variance homogeneity was assessed using Levene’s test. Statistical significance was set at a p-value threshold of less than 0.05.

## Results

In this part of the study, the findings obtained by analysing the data obtained as a result of this research conducted with 124 participants are presented. Information about the general characteristics of the participants in the Dietary Inflammatory Index quartiles is given in Table 2.

**Table 2:** General characteristics of participants in the dietary inflammatory index quartiles.

	Total		Q1		Q2		Q3		Q4		Test	*p*
General Characteristics	Mean	SD	Mean	SD	Mean	SD	Mean	SD	Mean	SD		
Age (years)	42.20	11.00	44.77	10.23	43.77	13.25	40.35	10.97	40.03	8.85	1.485^F^	0.222
Dİİ	1.44	1.23	−0.15	0.60	1.03	0.29	1.90	0.23	3.01	0.44	318.252^F^	**0.000**
**Gender**	***n***	**%**	***n***	**%**	***n***	**%**	***n***	**%**	***n***	**%**	**Test**	***p***
Female	75	60.5	21	16.9	17	13.7	18	14.5	19	15.3	1.181^χ²^	0.758
Male	49	39.5	10	8.1	14	11.3	13	10.5	12	9.7		
**Education status**												
Illiterate	10	8.1	1	0.8	0	0.0	4	3.2	5	4.0	17.744^χ²^	0.473
Literate	5	4.0	1	0.8	1	0.8	2	1.6	1	0.8		
Primary School	44	35.5	12	9.7	13	10.5	9	7.3	10	8.1		
Middle School	18	14.5	4	3.2	7	5.6	4	3.2	3	2.4		
High School	23	18.5	6	4.8	7	5.6	7	5.6	3	2.4		
University	26	21.0	7	5.6	5	4.0	5	4.0	9	7.3		
**Occupational status**												
Housewife	14	11.3	3	2.4	3	2.4	3	2.4	5	4.0	10.438^χ²^	0.917
Not working	14	11.3	3	2.4	4	3.2	2	1.6	5	4.0		
Officer	14	11.3	4	3.2	3	2.4	3	2.4	4	3.2		
Labourer	2	1.6	0	0.0	1	0.8	1	0.8	0	0.0		
Self-employment	60	48.4	18	14.5	14	11.3	16	12.9	12	9.7		
Student	15	12.1	2	1.6	4	3.2	6	4.8	3	2.4		
Pensioner	5	4.0	1	0.8	2	1.6	0	0.0	2	1.6		
**Marital status**												
Married	105	84.7	26	21.0	24	19.4	28	22.6	27	21.8	2.175^χ²^	0.537
Single	19	15.3	5	4.0	7	5.6	3	2.4	4	3.2		

*Q* Quartile, *F* One-way Anova test statistic, *χ²* Chi-square test statistic. Bolded values indicate statistical significance.

The mean age of the participants was 42.2 ± 11.0 years. 39.5% of the participants were male and 60.5% were female; 35.5% were primary school graduates, 18.5% were high school graduates and 18.5% were university graduates. While 12.1% of the participants were not working, 11.3% were civil servants, 48.4% were housewives, 84.7% were married and 15.3% were single. No significant relationship was found between the general characteristics of the participants and the quartile groups (*p* > 0.05).

The distribution of anthropometric, biochemical and sleep quality measurements of the participants according to quartile groups is given in Table 3.

**Table 3:** Distribution of anthropometric, biochemical and sleep quality measurements of the participants according to quartile groups.

	Q1		Q2		Q3		Q4		Test	*p*
Anthropometric Measurements	Mean	SD	Mean	SD	Mean	SD	Mean	SD		
Height length (cm)	162,52	9,06	164,74	7,43	163,77	7,29	164,70	7,49	0.550^F^	0.649
Body weight (kg)	90,38^a^	12,90	99,90^b^	14,29	98,40	11,51	97,15	14,62	3.057^F^	**0.031**
Body mass index (kg/m2)	34,12^a^	3,17	36,66^b^	3,25	36,66^b^	3,32	35,67	3,58	3.996^F^	**0.009**
Body fat percentage (%)	41.64	3.54	42.99	3.91	43.84	4.58	43.57	4.43	1.738^F^	0.163
Body muscle weight (kg)	48.13	6.93	51.93	6.30	51.75	7.24	50.04	5.22	7.28^χ²^	0.064
Body water content (%)	45.07^a^	3.72	42.80^b^	3.79	41.57^b^	4.10	41.71	4.75	15.31^χ²^	**0.002**
Waist circumference (cm)	107.26	8.49	111.07	9.12	112.10	8.22	110.97	9.10	6.54^χ²^	0.088
Hip circumference (cm)	113.81	12.67	116.10	12.72	115.61	11.88	115.52	9.63	0.223^F^	0.880
Waist/hip ratio	0.97	0.09	0.96	0.11	0.95	0.09	0.97	0.10	0.297^F^	0.827
Waist/height ratio	0.67	0.06	0.67	0.05	0.69	0.06	0.67	0.05	0.864^F^	0.462
**Biochemical Measurements**										
Fasting blood glucose (mg/dL)	94.10	6.21	92.58	6.56	93.74	5.32	92.74	5.41	0.493^F^	0.688
Serum triglyceride (mg/dL)	128.13	20.07	124.65	27.46	123.36	25.32	120.90	30.61	0.411^F^	0.745
HDL-cholesterol (mg/dL)	43.64	5.71	47.23	9.57	44.88	9.18	49.25	10.89	1.227^F^	0.307
LDL-cholesterol (mg/dL)	112.50	17.90	108.33	25.26	92.38	21.44	103.61	20.02	5.879^χ²^	0.118
Total cholesterol (mg/dL)	181.16^a^	19.04	178.45^a^	20.47	158.91^b^	39.19	173.71	19.02	10.275^χ²^	0.160
CRP (mg/l)	1.63^a^	2.66	3.05	4.43	5.98^b^	7.27	3.89	5.25	12.348^χ²^	**0.006**
Leukocytes (103/µl)	7.88	1.79	7.70	1.75	7.73	2.39	7.79	2.00	0.051^F^	0.985
Lymphocytes (103/µl)	2.58	0.90	2.61	0.80	2.33	0.79	2.44	0.71	0.777^F^	0.509
Neutrophils (103/µl)	4.51	1.25	4.35	1.15	4.61	1.92	6.72	11.12	1.191^F^	0.316
Eosinophils (103/µl)	0.18	0.16	0.18	0.12	0.14	0.08	0.16	0.11	0.644^F^	0.886
Monocytes (103/µl)	0.58	0.17	0.53	0.18	0.49	0.21	0.48	0.15	2.220^F^	0.089
**Sleep quality score**	7.90	2.57	6.61	3.13	7.36	3.12	6.94	2.94	1.109^F^	0.348
**Sleep quality status**	***n***	**%**	***n***	**%**	***n***	**%**	***n***	**%**	**Test**	***p***
Good	2	1.61	8	6.45	8	6.45	7	5.64	4.960^χ²^	0.175
Bad	29	23.39	23	18.55	23	18.55	24	19.35		

*Q* Quartile, *DII* Dietary inflammatory index, *SD* Standard deviation, *F* One-way Anova test statistic, *χ²* Kruskal-wallis test statistic, *χ²* Chi-square test statistic, *p* < 0.05. Bolded values indicate statistical significance.

It was found that as the inflammatory score of the diet consumed by the participants increased, body weight, BMI increased significantly and body fluid ratio decreased significantly (*p* < 0.05). In addition, CRP, one of the biochemical parameters, was found to increase significantly with the increase in the inflammatory score of the diet (*p* < 0.05).

Table 4 shows the vials for the comparison of anthropometric measurements, biochemical measurements, sleep quality scores and dietary ınflammatory ındex scores of the participants.

**Table 4:** Comparison of anthropometric measurements. biochemical measurements. sleep quality scores. and dietary ınflammatory ındex scores of the participants.

	Dietary Inflammatory Index Scores	
	*r*	*p*
Age	−0.194^p^	**0.031**
**Anthropometric measurements**		
Body weight (kg)	0.157^p^	0.082
BMI (kg/m^2^)	0.106^p^	0.242
Body fat percentage (%)	0.171^p^	0.058
Body muscle weight (kg)	0.094^p^	0.298
Body fluid ratio (%)	−0.260^p^	0.004^**^
Waist circumference (cm)	0.112^p^	0.217
Hip circumference (cm)	−0.010^p^	0.911
Waist/hip	−0.023^p^	0.796
Waist/height	−0.028^p^	0.758
**Biochemical measurements**		
Fasting blood glucose (mg/dl)	−0.110^p^	0.226
Serum triglyceride (mg/dl)	−0.076^p^	0.401
HDL-cholesterol (mg/dl)	0.061^p^	0.500
LDL-cholesterol (mg/dl)	−0.091^p^	0.313
Total cholesterol (mg/dl)	−0.243^S^	0.007**
CRP (mg/l)	0.258^S^	0.004**
Leukocytes (103/µl)	−0.036^p^	0.692
Lymphocytes (103/µl)	−0.114^p^	0.207
Neutrophils (103/µl)	0.180^p^	0.840
Eosinophils (103/µl)	−0.049^p^	0.587
Monocytes (103/µl)	−0.221^p^	0.013*
**Sleep Quality Score**	−0.073^p^	0.424

*BMI* Body Mass Index, *S* Spearman correlation, *P* Pearson correlation, *p < 0.05, **p < 0.01. Bolded values indicate statistical significance.

A negative weak significant correlation was found between the increase in age of the participants and DII (*p* < 0.05). A negative weak significant correlation was found between body water ratio, total cholesterol and monocyte level and DII score, while a positive weak significant correlation was found with CRP (*p* < 0.05).

## Discussion

In this pilot study involving 124 individuals with obesity, the relationship between the inflammatory index of diets consumed and inflammation and sleep quality problems in this population was examined.

Obesity is a complex health condition associated with increased levels of inflammation and poor sleep quality. In a study conducted by Göktaş et al. it was found that 81.3% of individuals with obesity had poor sleep quality [15]. In addition, in a study conducted by Altın et al. it was found that sleep quality worsened as the BMI of the participants increased and 75% of individuals with obesity experienced poor sleep quality [16]. The findings of this study, in line with previous studies in the literature, reveal that poor sleep quality is highly prevalent in people with obesity.

Dietary habits and culture significantly impact anthropometric measurements. Research examining the relationship between diet’s inflammatory score and anthropometric measurements found higher BMI and waist/hip ratios in individuals following pro-inflammatory diets [17]. Similarly, another study reported increased BMI, waist circumference, and waist/height ratios in those consuming a pro-inflammatory diet [18]. An investigation focusing on adults’ BMI and waist circumference in relation to their DII scores observed that those with higher DII scores had significantly larger waist circumferences, although no significant correlation with BMI was found [19]. In this study, it was found that the BMIs of those who were fed with lower DII scores were significantly lower and the findings of the study were found to be consistent with the literature.

In examining the association between DII scores and CRP, a key inflammatory marker, Shivappa et al. (2018) found a strong positive correlation [20]. This is echoed in a study by the Korea National Health and Nutrition Examination Survey (KNHANES, 2015), which showed a positive correlation between DII scores and CRP levels [21]. Similarly, a cross-sectional study involving 1992 adults revealed that higher DII scores were associated with elevated CRP values [19]. In the present study, the CRP levels of the groups fed with higher DII scores were found to be significantly increased and the findings of the study were found to be consistent with the literature.

The relationship between the DII and sleep quality has been a subject of varying research findings. Setayesh et al. (2021) identified a significant link between high DII scores and poor sleep quality, suggesting that diets with higher inflammatory potential may negatively impact sleep [22]. In contrast, a study focusing on university students by Masaad et al. (2021) did not find a statistically significant association between DII scores and sleep quality [23]. Interestingly, in this study, no significant association was found between the inflammatory score of the anti-inflammatory diet consumed and sleep quality. What is even more interesting in this study is that the consumption of diets with anti-inflammatory properties was not associated with sleep quality. However, a significant proportion of the study population had poor sleep quality, and as DII scores increased, a decline in sleep quality was noted, although not significantly. The simultaneous occurrence of high DII scores and increased poor sleep quality, especially in people with obesity, suggests that more detailed studies should be conducted on this subject.

### Strengths and limitations

The strengths of this study address an important health issue by analysing the relationships between DII, obesity, inflammation biomarkers and sleep quality based on current literature. The study controlled for variables that may affect the results by excluding individuals with chronic diseases. The study revealed a clear association between the consumption of diets with high DII scores and inflammatory markers and obesity. These findings provide valuable guidance for the development of targeted nutritional interventions, focusing on dietary factors associated with obesity. In addition to important findings, this study also has some limitations. Due to the small sample size, the significance of effects on individual parameters is limited; therefore, it was planned as a pilot study. The results obtained in this study, together with a review of the literature, reveal strong associations between dietary inflammatory load and critical determinants of human life such as age, BMI and inflammation. It was found that there is a relationship between the DII and sleep quality, although not directly, and that poor sleep quality increases with the presence of obesity.

## Conclusion

In this study involving people with obesity, an association was found between BMI and CRP and dietary inflammatory index scores. This association implies that dietary intake may influence the development of obesity through inflammatory pathways and that both obesity and inflammation may adversely affect humans. Consequently, it is imperative to promote the consumption of anti-inflammatory diets to counteract inflammation. Furthermore, it is recommended that future research should be strategically designed and conducted to examine in more depth the reciprocal relationships between obesity, inflammation, sleep quality and anti-inflammatory diets.

## Data Availability

Data used and/or analysed during the course of this study may be made available by the corresponding author upon reasonable request.
